# MicroRNA-15a/16/SOX5 axis promotes migration, invasion and inflammatory response in rheumatoid arthritis fibroblast-like synoviocytes

**DOI:** 10.18632/aging.103480

**Published:** 2020-07-17

**Authors:** Hua Wei, Qin Wu, Yumeng Shi, Aishu Luo, Shiyu Lin, Xiaoke Feng, Jintao Jiang, Miaojia Zhang, Fang Wang, Wenfeng Tan

**Affiliations:** 1 Division of Rheumatology, Clinical Medical College, Yangzhou University, Jiangsu Province, China; 2 Division of Rheumatology, The First Affiliated Hospital of Nanjing Medical University, Jiangsu Province, China; 3 Division of Rheumatology, The First People’s Hospital of Yancheng, Jiangsu Province, China; 4 Institute of Integrated Chinese and Western Medicine, Nanjing Medical University, Jiangsu Province, China; 5 Division of Cardiology, The First Affiliated Hospital of Nanjing Medical University, Jiangsu Province, China

**Keywords:** rheumatoid arthritis, miR-15a/16, SOX5, fibroblast-like synoviocytes

## Abstract

Fibroblast-like synoviocytes (FLSs) are key effector cells in the pathogenesis of rheumatoid arthritis (RA) and display a unique aggressive tumor-like phenotype with remarkable hyperplasia, increased cell migration and invasion. How FLSs undergo these changes in RA remains unknown. We previously reported a novel function of transcription factor SOX5 in RA-FLSs that promote cell migration and invasion. In this study, we found that miR-15a/16 directly targets the *SOX5* 3’UTR and suppresses *SOX5* expression. Moreover, miR-15a/16 is significantly down-regulated in RA-FLSs, which negatively correlates with *SOX5* expression. Transfection with miR-15a/16 mimics in RA-FLSs inhibits cell migration, invasion, *IL-1β* and *TNFα* expression. Overexpression *SOX5* in RA-FLSs decreases miR-15a/16 expression and rescues miR-15a/16-mediated inhibitory effect. Furthermore, RA patients with the lower baseline serum miR-15a/16 level present poor response of 3 months disease-modifying antirheumatic drugs (DMARDs) therapy. Collectively, this study reveals that miR-15a/16/SOX5 axis functions as a key driver of RA-FLSs invasion, migration and inflammatory response in a mutual negative feedback loop and correlates with DMARDs treatment response in RA.

## INTRODUCTION

Rheumatoid arthritis (RA) is the most common autoimmune disease affecting approximately 1% of the population worldwide. Persistent synovitis is the hallmark feature of RA, which leads to bone and cartilage destruction and subsequent functional disability in affected joint [1]. Fibroblast-like synoviocytes (FLSs) are the key effect cells contributing to chronic unresolved inflammation in synovial tissue of RA [2]. In RA, FLSs acquire a unique aggressive and tumor-like phenotype with remarkable hyperplasia, aggressive and invasive properties that perpetuate diseases and joint destruction [2]. How FLSs change in RA remains unclear. Exploring the controlling factors of FLSs not only provides novel insights into disease mechanisms, but also promotes the identification of new therapeutic strategy targeting FLSs in RA.

SOX5 (sex determining region Y-box protein 5) belongs to the family of SOX transcription factors [3]. *SOX5* gene is best-known to regulate embryonic development, cell fate determination and chondrogenesis [3]. In our previous studies, *SOX5* was overexpressed in RA-FLSs compared with osteoarthritis (OA) FLSs. By binding to the promoter of *MMP9*, it played a crucial role in promoting the migration and invasion of FLSs [4]. According to robust data from our previous studies, SOX5 promotes an aggressive biological behavior in RA-FLSs, but the mechanism remains unknown.

MicroRNAs (miRNAs) are small non-coding RNAs (21-25 nucleotides) that regulate gene expression at the posttranscriptional level by directly binding to the 3'-untranslated region (UTR) of target mRNA [5]. More than 2000 miRNAs have been identified in humans, emerging as important controllers of many cellular events [6]. Some of these miRNAs attract special attention for modulating certain target gene expression to regulate FLSs behaviors. In RA synovial tissue and RA-FLSs, significantly down-regulated miR-19 [7], miR-92a [8], miR-650 [9] and miR-613 [10] respectively target *TLR2*, *AKT2* and *DKK1*, inhibiting FLSs proliferation and migration. miR-18a [11], miR-19 [12], miR-21 [13], miR-155 [14], miR-146 [15] and miR-663 [16] are overexpressed at synovial tissue, promoting inflammation, cell migration and invasion of RA-FLSs by interacting with the NF-κB and Wnt signaling pathway. Thus, we hypothesize that miRNAs may interact with *SOX5* to modulate migration and invasion of FLSs.

We identified *SOX5* as a potential target gene of miR-15a and miR-16 using TargetScan database. miR-15a and miR-16 belong to the miR-15 family together with miR-15b, miR-195, miR-424, and miR-497 [17]. miR-15a and miR-16 cluster at chromosomal location 13q14 and possess the same seed sequence with similar biological functions [18]. The miR-15a/16 cluster was first considered a tumor suppressor regulating cell differentiation, proliferation, maturation, apoptosis or angiogenesis in several types of human cancer [19–21]. Recently, growing evidence indicates that miR-15a/16 regulates multiple immune processes including NK maturation [22], T cell development [23] and macrophage phagocytosis after bacterial infection [24]. The role of miR-15a/16 in RA remains elusive.

In this study, we demonstrate that miR-15a/16 and *SOX5* form a mutual negative feedback regulatory loop, which drives pathological behavior of RA-FLSs including cell migration, invasion and inflammatory response. Moreover, we also reveal that serum miRNA-15a/16 could serve as a potential biomarker for predicting DMARDs therapy response in RA patients.

## RESULTS

### Reciprocal repression between miR-15a/16 and SOX5

**Figure 1:**
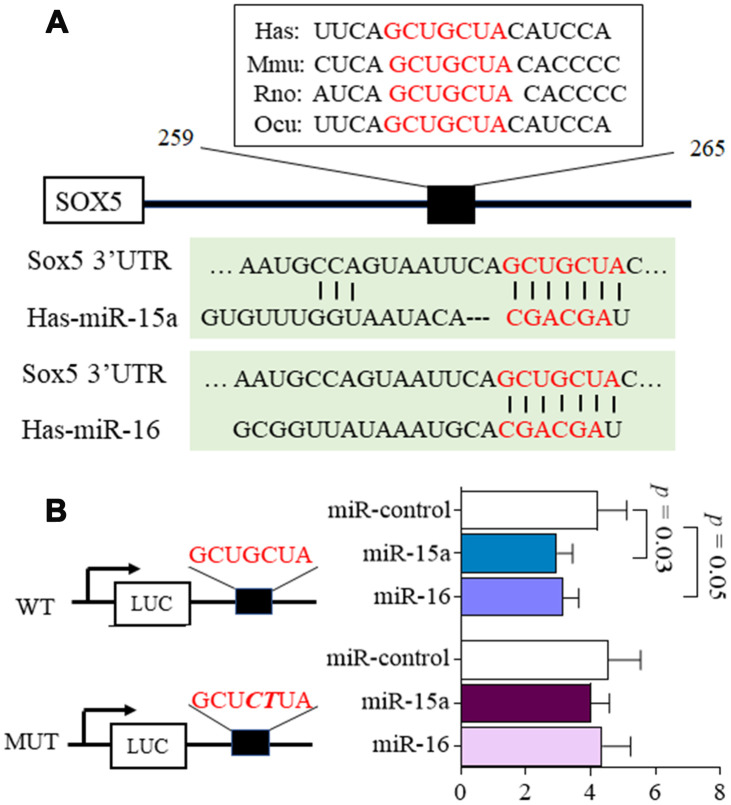
**MicroRNA-15a/16 targets the 3’UTR of *SOX5*.** (**A**) Schematic representation of the putative target site for miR-15a/16 in the 3’UTR of *SOX5*. The binding sites of miR-15a/16 in *SOX5* 3’UTR were conserved among species. (**B**) 293T cells were co-transfected with luciferase reporter containing wild-type (WT), mutant (MUT) 3’UTR of *SOX5* and miRNA mimics. Mutations within the seed sequence were marked as bold italic. After transfection for 48h, the luciferase intensity was measured and renilla intensity was used as for normalization. Bars show the mean ± SD of 3 independent experiments.

Using Target Scan 7.2 (http://www.targetscan.org/), we predicted that the putative miR-15a/16 target site should be located within the position 259-265 of *SOX5* 3’UTR. This binding sites in *SOX5* 3’UTR were conserved among species (Figure 1A). To test whether *SOX5* is a functional target of miR-15a/16, the *SOX5* 3’UTR containing wild type (WT) and mutant (MUT) miR-15a/16 binding sites were subcloned into the luciferase reporter vector. Co-transfection with the WT *SOX5* 3’UTR and miR-15a/16 mimic into 293T cell for 48hrs significantly decreased the luciferase intensity of *SOX5* 3’UTR (Figure 1B). In contrast, co-transfecting with the mutant *SOX5* 3’UTR and miR-15a/16 mimic displayed no effect on luciferase activity (Figure 1B).

**Figure 2:**
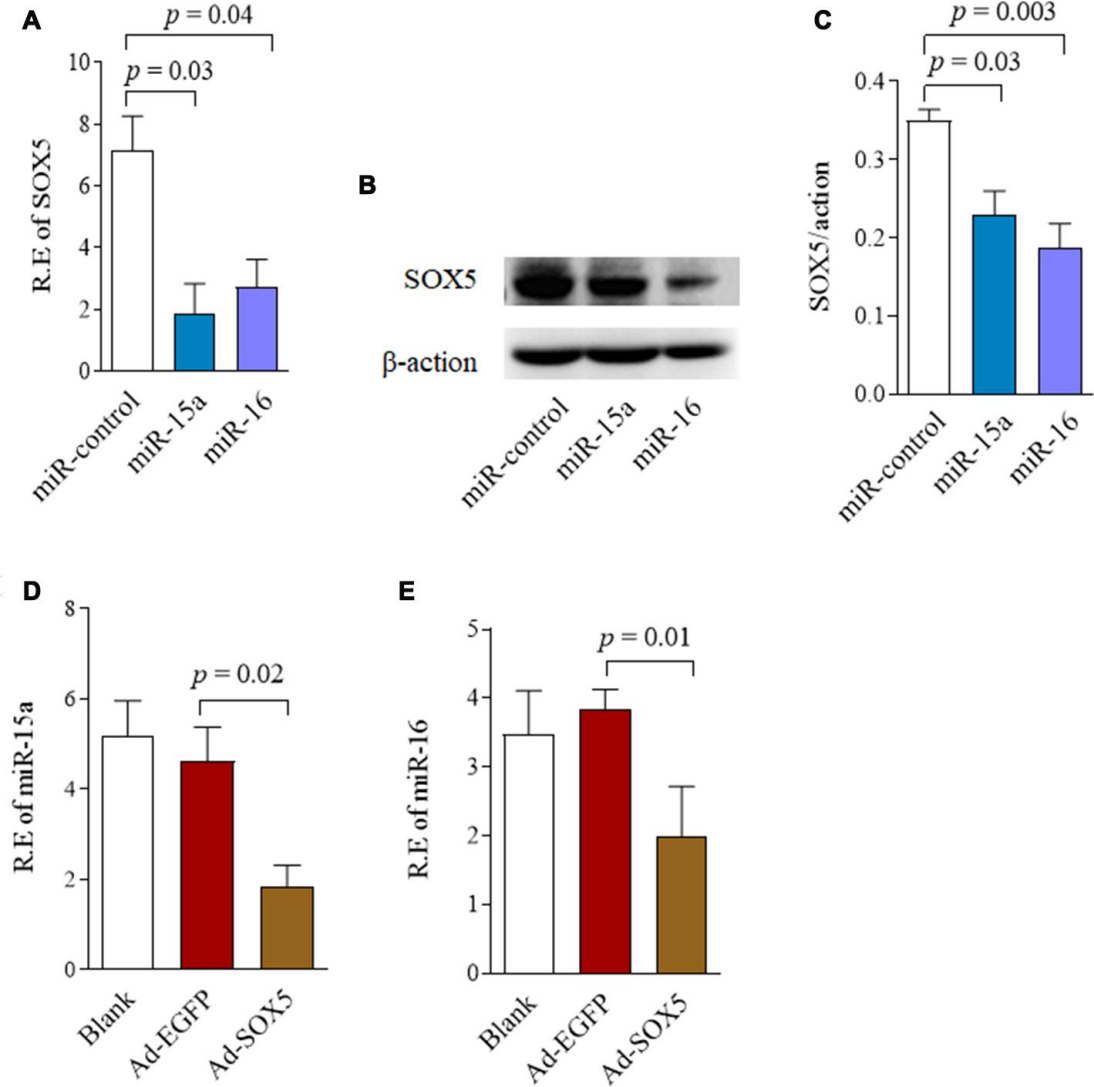
**Reciprocal repression between SOX5 and miR-15a/16.** (**A**, **B**) RA-FLSs cell line of MH7A was transfected with miR-15a/16 mimics for 48h. SOX5 expression level was determined by RT-qPCR (**A**) and western-blot (**B**). (**C**) Graphs show the quantitation data derived from left western-blot figure. (**D**, **E**) MH7A was transfected with Ad-SOX5 for 48h. The expressions of miR-15a (**D**) and miR-16 (**E**) were detected by RT-qPCR. Bars show the mean ± SD of 3 independent experiments.

We then assessed the capability of miR-15a/16 to negatively regulate *SOX5* expression. RA-FLSs cell line of MH7A was transfected with miR-15a/16 mimics for 48hrs. Endogenous SOX5 expression were then detected by RT-qPCR and western-blot. As shown in Figure 2, the mRNA (Figure 2A) and protein levels (Figure 2B, 2C) of SOX5 markedly decreased, followed by miR-15a or 16 overexpression compared with the miR-control transfection. Taken together, our data suggest that miR-15a/16 could directly bind to *SOX5* 3’UTR and inhibit SOX5 expression, implying a posttranscriptional mechanism of regulating *SOX5* in RA-FLSs.

To determine whether miR-15a/16 is regulated by *SOX5*, we detected miR-15a/16 expression after *SOX5* overexpressed in FLSs by transfecting with Ad-SOX5 into MH7A. *SOX5* overexpression significantly decreased miR-15a (Figure 2D, *p* = 0.02) and 16 (Figure 2E, *p* = 0.01) expression levels in MH7A compared with the control groups. These data suggested that there might be a reciprocal repression between *SOX5* and miR-15a/16.

### Expression of miR-15a/16/SOX5 axis in primary FLSs from RA patients

**Figure 3:**
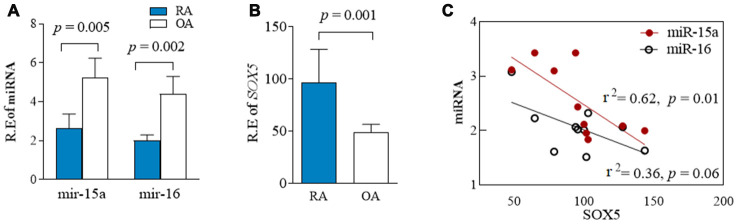
**Expression of miR-15a/16/SOX5 axis in primary FLSs from RA and OA patients.** (**A**, **B**) Expression of miR-15a/16 (**A**) and *SOX5* (**B**) in primary FLSs from RA (n = 10) and OA (n = 10) patients was detected simultaneously by RT-qPCR. (**C**) Correlation between the expression of miR-15a/16 and *SOX5* mRNA. Bars show the mean ± SD.

We previously reported higher SOX5 expression in synovial tissue of RA patients than those in OA patients [4]. To further ascertain the relationship between miR-15a/16 and *SOX5* expression in RA patients, we therefore used RT-qPCR to simultaneously examine miR-15a/16 and *SOX5* expression in primary FLSs of RA and OA patients. The expression of miR-15a and 16 significantly decreased (*p* = 0.005 and 0.002, respectively, Figure 3A), while *SOX5* levels (*p* = 0.001, Figure 3B) markedly increased in RA-FLSs (n = 10), as compared with those of OA patients (n = 10). A strong negative correlation existed between the expression levels of miR-15a and *SOX5* in RA-FLSs (*p* = 0.01, r^2^ = 0.62, Figure 3C). A similar negative correlation trend was also found between miR-16 and *SOX5*, but did not reach the statistical difference (*p* = 0.06, r^2^ = 0.36, Figure 3C). These data suggest the down-regulated miR-15a might link to an increase in its target gene *SOX5* expression in RA-FLSs, supporting that the negative feedback loop between miR-15a/16 and *SOX5* axis participates in RA pathogenesis.

### Effect of miR-15a/16/SOX5 axis on RA-FLSs migration and invasion

**Figure 4:**
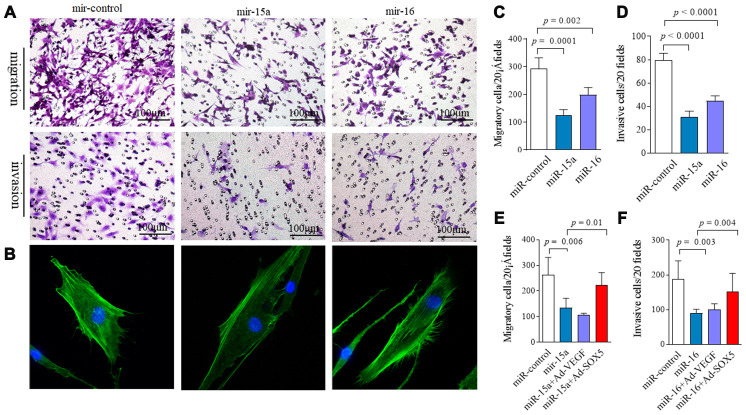
**Regulation of miR-15a/16/SOX5 axis on RA-FLSs migration and invasion.** (**A**) Following transfected with miR-15a, miR-16 mimics and miR-control for 48h, FLSs subjected to transwell (**A**, above) and transwell chamber invasion assay (**A**, below) after 24h. (**B**) Following transfected with miR-15a, miR-16 mimics and miR-control for 48h, FLSs were fixed and stained with FITC-phalloidin. Representative confocal microscopy images of three independent experiments are shown to illustrate stress fibers and appearance of lamellipodia. (**C**, **D**) Graphs show the quantitation data derived from the left figure **A**. Data are each representative of 3 independent experiments. (**E**, **F**) *SOX5* overexpression alleviates the miR-15a/16 mimics-mediated inhibitory roles on migration (**E**) and invasion (**F**) in FLSs. Graphs show the quantitation data derived from 3 independent migration and invasion assay.

In our previous data, SOX5 showed the potent ability to prompt migration and invasion of RA-FLSs [4]. Thus, we sought to determine whether lower expression of miR-15a/16 contributes to the pathologic behavior in RA-FLSs, and whether it does so via *SOX5*. MH7A were transfected with miR-15a or miR-16 mimics for 48hrs. As shown in Figure 4, overexpression of miR-15a and 16 in RA-FLSs decreased cell migration by 60% (*p* = 0.0001) and 34% (*p* = 0.002) respectively compared with the controls (Figure 4A above and Figure 4C). Similarly, an in vitro invasion assay showed that miR-15a and 16 mimics significantly inhibited invasiveness of RA-FLSs by 62% and 43%, respectively (Figure 4A below and Figure 4D). Consistent results of miR-15a/16 overexpression suppressing cell migration and invasion were validated in primary RA-FLSs (n=3) (Supplementary Figure 1).

Cell migration and invasion are commonly accompanied by dynamic reorganization of the actin cytoskeleton. We examined the rearrangements of actin cytoskeleton in RA-FLSs after transfection with miR-15a/16 using F-actin staining. Compared with the controls (Figure 4B), miR-15a mimics markedly decreased stress fibers’ appearance and lamellipodia formation in RA-FLSs. However, miR-16 overexpression did not markedly affect the cytoskeletal reorganization in RA-FLSs (Figure 4B). Cell proliferation was also measured by CCK-8 assay. miR-15a and miR-16 overexpression had no effect on cell proliferation at each time point from 0 to 72hrs (data not shown).

The data above suggest that overexpression miR-15a/16 resulted in an inhibitory role on FLSs. We then performed rescue experiments. Following transfecting with Ad-SOX5 into MH7A for 72hrs, low migration (Figure 4E) and invasion of FLSs (Figure 4F) by miR-15a/16 mimics transfection was markedly alleviated by *SOX5* overexpression. Taken together, these data suggest that miR-15a/16/SOX5 axis participated in driving RA-FLSs migration and invasion.

### Regulation of miR-15a/16/SOX5 axis on cytokine production in RA-FLSs

**Figure 5:**
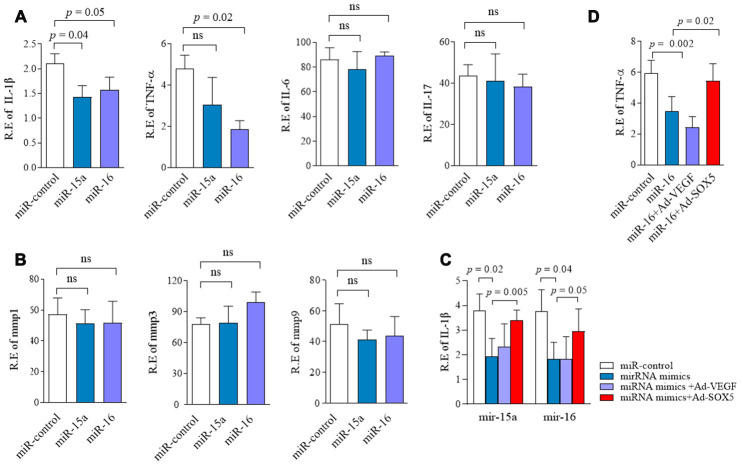
**Regulation of miR-15a/16/SOX5 axis on cytokine production in RA-FLSs.** (**A**, **B**) Following transfected with miR-15a, miR-16 mimics and miR-control for 48h, expression of *IL-1β,* *TNF-α*, *IL-6, IL-17* (**A**), *MMP-1*, *MMP-3* and *MMP-9* (**B**) was detected by RT-qPCR. (**C**, **D**) *SOX5* overexpression alleviates the miR-15a/16 mimics-mediated inhibitory roles on *IL-1β* (**C**) and *TNF-α* (**D**) expression in FLSs. Bars show the mean ± SD of 3 independent experiments.

Since SOX5 was involved in regulating IL-6, MMP-3, and MMP-9 expression in RA-FLSs in our previous studies [4], we subsequently tested the effect of miR-15a/16/SOX5 axis on inflammatory response. In addition to *IL-6*, *MMP-3*, and *MMP-9,* a series of cytokines, including *IL-1β*, *IL-17*, *TNF-α*, and *MMP-1*, which were highly expressed in RA synovial tissues and contributed to the invasive ability of RA-FLSs [2]. Expression of these cytokine were detected by RT-qPCR in RA-FLSs. Overexpression of miR-15a markedly inhibited *IL-1β* production (*p* = 0.04) in FLSs but did not affect any other cytokines expressions (Figure 5A, 5B). miR-16 mimics showed the ability to significantly suppress *IL-1β* (*p* = 0.05) and *TFN-α*(*p* = 0.02) expression in RA-FLSs compared with miR-control transfection group (Figure 5A, 5B). These data indicated that miR-15a/16 could decrease cytokines production in RA-FLSs. Furthermore, miR-15a/16 mimics-medicated lower *IL-1β* or *TFN-α* expression could also be blocked by *SOX5* overexpression (Figure 5C, 5D). In primary RA-FLSs, SOX5 also showed the ability to rescue the lower *IL-1β* and *TFN-α* expression was caused by miR-15a/16 mimics transfection (Supplementary Figure 2). These results suggested a crucial role of miR-15a/16/SOX5 in the inflammatory process in RA-FLSs.

### Association miR-15a/16 with treatment response in RA patients

We previously failed to identify the significant correlation between serum SOX5 levels and RA disease activity score of DAS28. We then addressed whether miR-15a/16 could function as a biomarker for predicting RA disease activity or therapy response. We first examined the expression miR-15a/16 in synovial tissues from responders and non-responders of DMARDs therapy (non-responders are defined as DAS28>3.2 after more than three months of DMARDs treatment). Expressions of miR-15a and 16 decreased more significantly in non-responders than those in responders (p = 0.03 and 0.04, respectively, Supplementary Figure 3), implying that miR-15a and 16 levels in synovial tissues might be related to therapy response.

**Figure 6:**
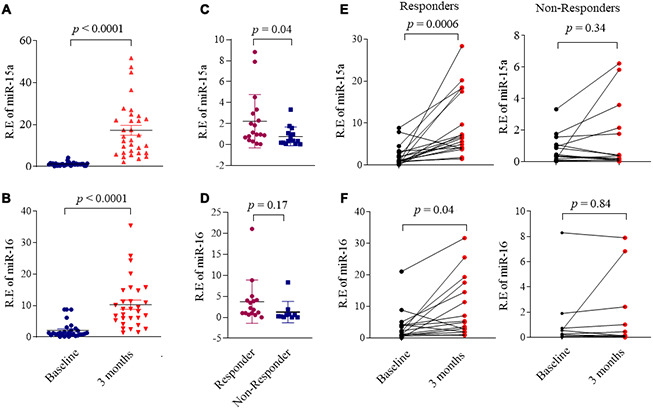
**Association miR-15a/16 with DMARDs treatment response in RA patients.** (**A**, **B**) Expression of miR-15a (**A**) and miR-16 (**B**) in serum from RA patients (n = 32) at baseline and after 3 months DMARDs therapy. (**C**, **D**) RA patients were divided RA into responders and non-responders based on whether their DAS28 score was changed ≥ 1.2 after 3 months DMARDs therapy. Graphs show the serum levels of miR-15a (**C**) and miR-16 (**D**) in serum from responders (n = 18) and non-responders (n=14). (**E**, **F**) Changes of the serum levels miR-15a (**E**) and miR-16 (**F**) in responders (left) and non-responders (right) from baseline to after 3 months DMARDs therapy. Values are the mean ± SD.

An ideal biomarker should be obtained easily. We therefore tested the circulating miR-15a/16 levels in serum from DMARDs naïve RA patients and HC. The baseline serum miR-15a/16 expression levels in RA showed neither the statistical difference with those in HC serum nor the strong association with RA disease activity score of DAS28 (data not shown). However, we found that miR-15a/16 levels significantly increased after 3 months of DMARDs therapy (Figure 6A, 6B) as compared with baseline, following up the average DAS28 decreased from 5.1 at baseline to 3.6 at 3 months. We divided RA patients into responders and non-responders based on whether their DAS28 score has changed ≥1.2 after 3 months of DMARDs therapy. We demonstrated that the baseline circulating miR-15a levels was higher in responders than those in non-responders (*p* = 0.04) (Figure 6C). The baseline miR-16 levels also increased in responders as compared with non-responders, but did not reach statistical significance (Figure 6D). Accompanied by the decreased disease activity at 3 months after DMARDs treatment, serum miR-15a (Figure 6E, left) and miR-16 (Figure 6F, left) levels increased significantly from baseline to 3 months (*p* = 0.0006 and 0.04, respectively) in responders; however, the non-responders retained similar low miR-15a/16 levels at 3 months as compared to baseline (p = 0.34 and 0.84, respectively) (Figure 6E, 6F, right). These data suggest that low serum levels of miR-15a/16 at baseline are associated with inadequate response of 3 months DMARDs treatment in RA patients.

## DISCUSSION

SOX5 is traditionally known as a regulator on embryonic development, cell fate determination and chondrogenesis [3]. We previously demonstrated SOX5’s critical role in regulating migration and invasion of RA-FLSs [4]. In this study, we demonstrate that miR-15a/16 directly targets the 3’-UTR of *SOX5*. *SOX5* and miR-15a/16 may consist of a negative feedback loop, promoting the migration, invasion and inflammatory response of RA-FLSs. Moreover, we also reveal that the lower baseline serum miR-15a/16 level in RA patients might be associated with poor response in 3 months of DMARDs therapy. These findings reveal a critical role of miRNA-15a/16/SOX5 axis in RA pathogenesis.

Down-regulation of miR-15a/16 has been identified in chronic lymphocytic lymphoma, prostate cancer, gastric cancer, non-small cell lung and osteosarcoma [19–21]. Low miR-15a/16 expression promotes cancer cell proliferation, migration and invasion, which makes it a tumor suppressor. [14–16]. Given RA-FLSs’ “tumor-like” features [25], it is not surprised to observe that miR-15a/16 negatively regulates RA-FLSs migration and invasion. miR-15a/16 exerts tumor suppressive roles by targeting multiple oncogenes, including *BCL2, TWIST1, MCL1, CCND1* and *WNT3A* [19–21]. According to our observation, miR-15a/16 decreased in RA-FLSs, while *SOX5* expression level markedly increased. We reveal that miR-15a/16 negatively regulates *SOX5* expression by directly targeting its 3’-UTR; furthermore, overexpressed, *SOX5* could lead to a decrease in miR-15a/16 expression supporting a negative regulatory feedback loop in RA-FLSs between miR-15a/16 and *SOX5*.

As a transcriptional factor, SOX5 can target multiple genes including *COL2A1*, *SPARC*, *TWIS*T and *RORγt* [26–29] (9), and participates in regulating chondrogenesis, tumor progression and Th17 cell differentiation. We previously revealed a critical role of *SOX5* in RA progression: it binds to the promoter of *RANKL* to prompt osteoclast activity [30] and targets on *MMP-9* promoter to enhance aggressive behavior in FLSs [4]. The current study indicates that in this negative feedback loop of miR-15a/16/SOX5, downregulation of miR-15a/16 could be one of the critical steps contributing to *SOX5* overexpression and then RA pathogenesis.

The mechanisms responsible to the downregulation of miR-15a/16 expression in RA-FLSs remains unclear. Loss of miR-15 and miR-16 has been described as a key event in cancer progression in different tumor types [31–33]. Point mutations in miR-15a/16 sequence might inactivate their expression in these tumors [19, 34, 35]. It can be speculated that genetic mutations partially are involved in less miR-15a/16 expression in RA-FLSs. Additionally, we prove that proinflammatory cytokines of IL-1β, TNF-α and IL-6 could induce up-regulation of *SOX5* in RA-FLSs [30]. It is possible that inflammatory microenvironment could exert an indirect role on lowered miR-15a/16 expression by inducing *SOX5* expression and triggering the negative feedback loop of miR-15a/16/SOX5 axis. Indeed, we cannot rule out the possibility that miR-15a/16 targets other genes involved in disease progression. In our previous study, SOX5 could bind to the promoter of *RANKL* and *MMP9* genes. However, we failed to identify the interaction between miR-15a/16 with them (data not shown).

Serum miR-15a/16 have been reported as a useful biomarker for distinguishing sepsis from systemic inflammatory response [36] and predicting prognosis in chronic lymphocytic leukemia [37]. Interestingly, after DMARDs therapy, miR-15a and 16 expression decreased more significantly in synovial tissue of non-responders than those in responders, prompting us to explore whether serum miR-15a/16 could serve as a potential predictive marker for disease activity or therapy response in RA. We failed to identify the correlation between miR-15a/16 downregulation and disease activity. Other compartments or mechanisms might link to it. However, we have demonstrated that the lower baseline expression of serum miR-15a/16 is associated with an insufficient response after 3 months of DMARDs therapy in RA patients. Given our data supported that miR-15a/16/SOX5 axis linked to the aggressive behavior in RA-FLSs, it is not surprising to observe the predictive role of miR-15a/16’s circulating levels on therapy response in RA.

In summary, our data provide evidence that a microRNA-15a/16/SOX5 axis plays a crucial role in regulating pathologic behavior in RA-FLSs. Further study needs to uncover the interacting mechanism involved in this feedback loop. Considering miR-16-based mimic microRNA has been used in recurrent malignant pleural mesothelioma therapy in a phase 1 clinical trial [38], these studies would provide the basis for the development of new miR-15a/16-targeted diagnosis and therapies for RA.

## MATERIALS AND METHODS

### Patients

A total of 32 newly diagnosed RA patients were included in this study. RA patients and healthy controls (HC, n = 20) were recruited from the First Affiliated Hospital of Nanjing Medical University. The criteria for patient eligibility were: (i) fulfillment of the revised 1987 American Rheumatism Association criteria for RA; (ii) naïve to DMARDs or biologics treatment; (iii) with active disease (defined as Disease Activity Score of 28 joints [DAS-28] > 3.2). This study was approved by the First Affiliated Hospital of Nanjing Medical University Committee on Human Research. All subjects in this study provided written informed consent. Clinical characteristics of RA and HC were shown in Supplementary Table 1.

### Cell culture

Human rheumatoid fibroblast-like synoviocytes MH7A cells used in this study were a generous gift from Dr. Seiichi Tanuma (Tokyo University of Science). MH7A cells were isolated from the intra-articular soft tissue of knee joints of RA patients and were established as a cell line by transfection with the SV40 T antigen. Primary RA FLSs samples were obtained from synovium biopsy and OA FLSs were derived from arthroplasty. Written consent was signed by these patients. MH7A and RA/OA-FLSs cells were respectively cultured in 1640 and DMEM medium supplemented with 10% fetal bovine serum (Gibco, Carlsbad, CA, USA) 100 U/mL penicillin and 100 ug/mL streptomycin (Sigma Aldrich, St. Louis, MO, USA) at 37°C in a humidified atmosphere of 5% CO_2_ in air.

### Plasmid construction and luciferase reporter assay

MiR-15a/16 mimics and control were purchased from GenePharma (Shanghai, China). *SOX5* 3’UTR region was amplified from 293T cell genomic DNA, and the PCR product was cloned into pmiR-RB-ReportTM dual-luciferase reporter vector (Ribobio, Guangzhou, China) to generate *SOX5* 3’UTR wild-type (WT) and mutant plasmids. 293T cells were co-transfected with luciferase reporter constructs and WT or mutated corresponding *SOX5* using Lipofectamine 2000 DNA Transfection Reagent (Thermo Fisher Scientific, Cleveland, OH, USA), according to the manufacturer's protocol. After transfection for 48 hours, the activity of luciferase constructs was measured using a DualGlo Luciferase Assay System (Promega) according to the manufacturer’s instructions. The firefly luciferase activity was used for normalization. For construction of *SOX5* overexpression vector, the full-length of *SOX5* cDNA was generated via standard PCR and inserted into the pAd- CMV-MC5 as described (Ad-SOX5) [4].

### Cell proliferation, migration and invasion assay

MH7A cells were plated at a density of 8 × 10^3^ cells/well in a 96-well culture plate. miR-15a/16 mimics and control were transfected into MH7A using Lipofectamine 2000. The viability of cells was assessed by the Cell Counting Kit-8 (CCK-8; Dojindo China Co., Ltd., Shanghai) after transfecting for 24h, 48h and 72h, respectively. After transfecting with miR-15a/16 mimics and control for 48h, 5×10^4^ MH7A cells were placed into the chamber (8μm pore size, Corning, NY) with or without pre-coated with 50ul 1:5 diluted Matrigel (BD, San Jose, CA, USA). The transfected cells were resuspended in serum-free media and seed on the upper chamber for 24h, and the lower chamber were filled with complete media. Cells transmigrated into lower wells were fixed and stained with 0.1% crystal violet. The number of migrated cells was averaged from five 10×field-of-view images and normalized to control.

### Immunofluorescent staining for F-actin

Transfected FLSs were resuspended and seeded into 35mm confocal dish for 24h. Dishes were washed with PBS and fixed with 4 % paraformaldehyde in PBS for 20 mins. To observe F-actin, cells were stained with 5ug/ml FITA- phalloidin (Sigma, St. Louis, MO, USA). Nuclei were co-stained with 6-diamidine-20-phenylindole dihydrochloride (DAPI) (Sigma, St. Louis, MO, USA). Stained cells were examined and photographed using a laser scanning confocal microscope.

### Real-time quantitative PCR (qPCR)

For miRNAs expression, RNAs were extracted from serums using mirVanaTM Protein and RNA Isolation System (Invitrogen, Carlsbad, CA, USA), according to the manufacturer’s instructions. miRNAs were reverse transcribed using specific stem-loop miRNAs primers. About 300ng of RNA was used for reverse transcription. Small nuclear RNA U6 was used for normalization. RT-qPCR was performed using an Applied Biosystems 7900HT Fast Real-Time PCR System. For mRNA expression, total RNAs were extracted from primary FLSs or transfected cells using Trizol agent (Invitrogen) according to the manufacturer’s instructions. Total RNA was converted to cDNA using the Superscript II reverse transcriptase (Invitrogen). Primers used to amplify interest genes were listed in Supplementary Table 2. The measurement of specific gene expression was performed using the ABI Prism 7900 Sequence Detection System and analyzed using the ABI Prism 7900 SDS Version 1.0 software. Relative expressions of interest genes were normalized to β-actin and calculated by the 2^-ΔΔCt^ method.

### Western blotting assay

Western blotting was performed using whole cell lysates. Non-specific interactions were blocked with 5% skim milk for 2 hours and were then probed with phospho-p38, phospho-ERK, and phospho-JNK and then were re-probed with antibody against total p38, ERK, JNK (Cell Signaling Technology, Danvers, MA, USA) and SOX5 (Abcam, Cambridge, MA, USA). β-actin (Cell Signaling Technology, Danvers, MA, USA) was used as a protein loading control. Protein bands were visualized with Super Signal West Dura chemiluminescent detection reagents following the manufacturer's directions.

### Statistical analyses

All experiments were performed in triplicate. Results were expressed as the mean ± standard deviation (SD). The statistical significance of difference between an experimental group and its corresponding control was evaluated by a non-parametric Mann-Whitney U test (GraphPad Prism 8). The correlation between the relative expression levels of miR-15a/16 and *SOX5* was analyzed using Spearman’s ρ-test. *p* < 0.05 was considered statistically significant.

## Supplementary Material

Supplementary Figures

Supplementary Tables
