# Supplementary material for: MicroRNA-15a/16/SOX5 axis promotes migration, invasion and inflammatory response in rheumatoid arthritis fibroblast-like synoviocytes
Source: Aging (Albany NY). 2020 Jul 17;12(14):14376–90. doi: 10.18632/aging.103480 (PMC7425471; doi:10.18632/aging.103480)
Supplement: Supplementary Figures [file aging-12-103480-s002..pdf]

SUPPLEMENTARY FIGURES

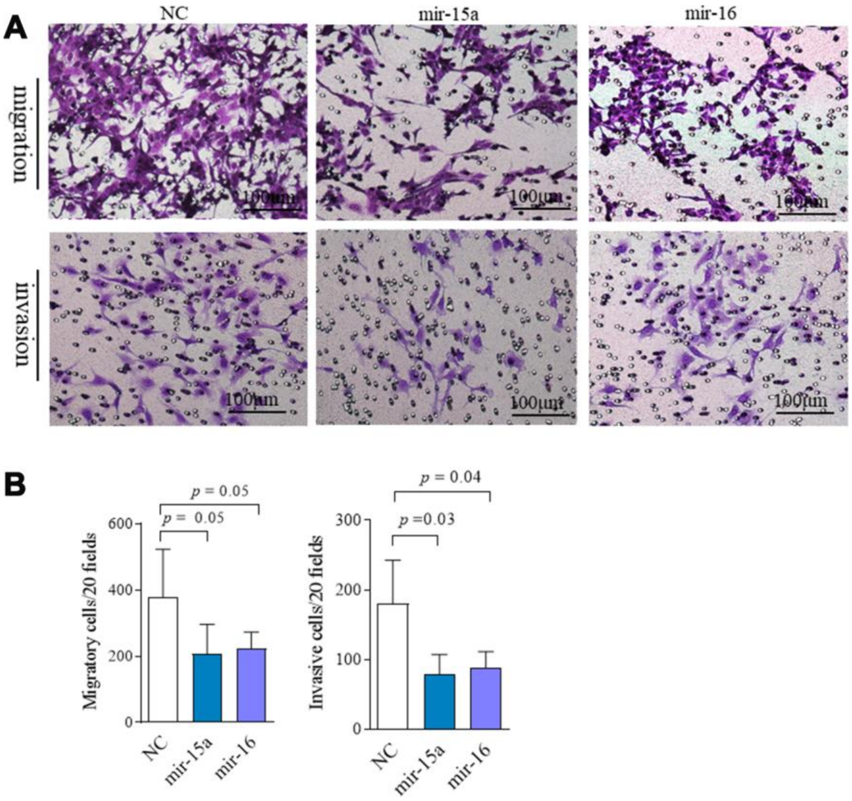

Supplementary Figure 1. Regulation of miR-15a/16/SOX5 axis on RA-FLS migration and invasion. Following transfected with miR-15a, miR-16 mimics and miR-control for 48h, primary RA-FLS subjected to transwell (**A**, above) and transwell chamber invasion assay (**A**, below) after 24h. (**B**) Graphs show the quantitation data derived from the left figure **A**. Data are each representative of 3 independent experiments.

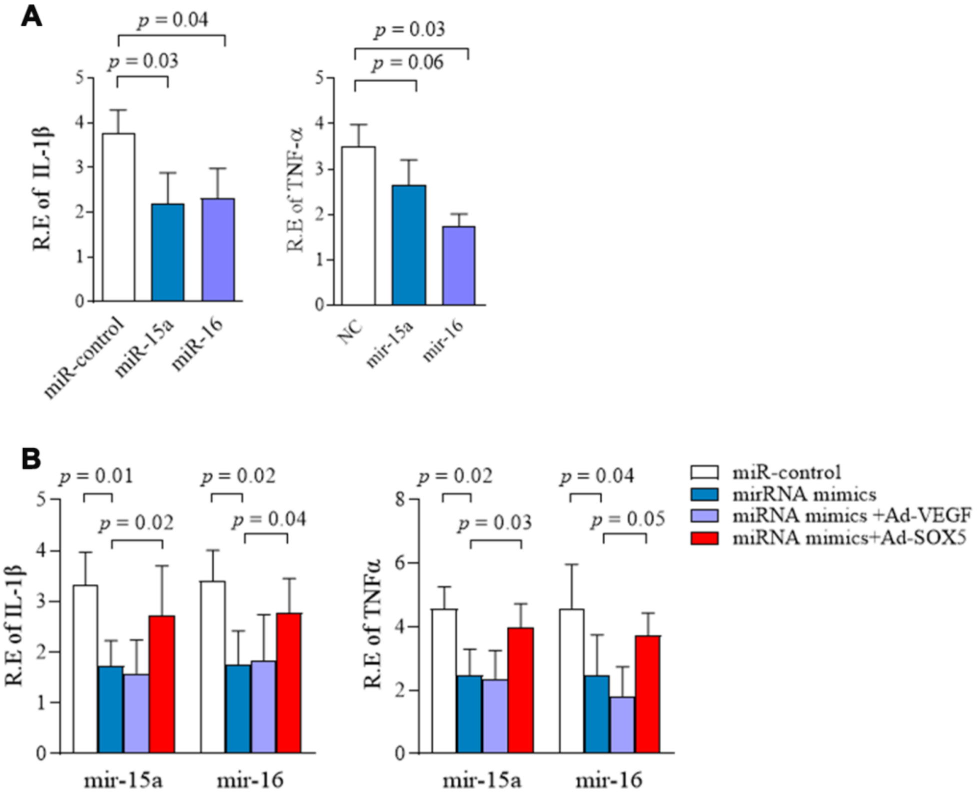

Supplementary Figure 2. Regulation of miR-15a/16/SOX5 axis on cytokine production in primary RA-FLS. (**A**) Following transfected with miR-15a, miR-16 mimics and miR-control for 48h, expression of *IL-1β* and *TNF-α* was detected by qRT-PCR. (**B**) *SOX5* overexpression alleviates the miR-15a/16 mimics-mediated inhibitory roles on *IL-1β* and *TNF-α* expression in FLS. Bars show the mean±SD of 3 independent experiments.

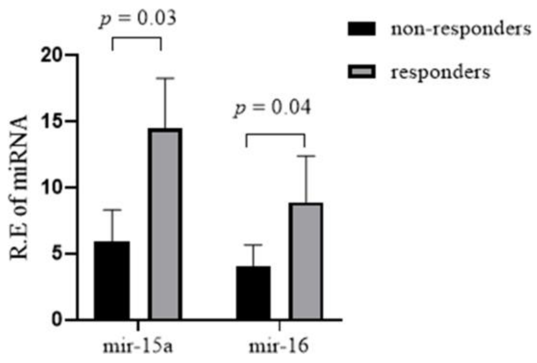

Supplementary Figure 3. Expression of miR-15a miR-16 in synovial tissues from responders (n=10) and non responders (n=10) of DMARDs therapy (non-responders are defined as DAS28>3.2 after more than three months DMARDs treatment).
