# Supplementary material for: MicroRNA-15a/16/SOX5 axis promotes migration, invasion and inflammatory response in rheumatoid arthritis fibroblast-like synoviocytes
Source: Aging (Albany NY). 2020 Jul 17;12(14):14376–90. doi: 10.18632/aging.103480 (PMC7425471; doi:10.18632/aging.103480)
Supplement: Supplementary Tables [file aging-12-103480-s001..pdf]

SUPPLEMENTARY TABLES

Supplementary Table 1.Demographic and clinical characteristics of RA patients and HC.

Parameter	RA (n=32)	HC (n=20)
Age	45.3±9.6	44.6±8.3
Female,%	89.1	91.5
Duration of symptoms, weeks	24.4±10.6	
Rheumatoid factor positive, %	70.5	
Anti-CCP positive, %	67.1	
DAS28	5.1±0.8	
Swollen joint count	12.1±5.3	
ESR (mm/h)	39.5±10.7	
CRP(mg/L)	18.1±10.1	

Supplementary Table 2. Human primers used for real-time quantitative PCR.

Gene Human	primer sequence
β-actin	sense 5′ CCACACTGTGCCCATCTACG 3′<br>anti-sense 5′ AGGATCTTCATGAGGTAGTCAGTCAG 3′
IL-6	sense 5′ AACCTGAACCTTCCAAAGATGG 3′<br>anti-sense 5′TCTGGCTTGTTCCTCACTACT 3′
IL-17	sense 5′ GGGCCTGGCTTCTGTCTG 3′<br>anti-sense 5′AAGTTCGTTCTGCCCCATCA 3′
IL-1β	sense 5′ATGATGGCTTATTACAGTGGCAA 3′<br>anti-sense 5′GTCGGAGATTCGTAGCTGGA 3′
TNF-α	sense 5′CCTCTCTCTAATCAGCCCTCTG 3′<br>anti-sense 5′GAGGACCTGGGAGTAGATGAG 3′
MMP-1	sense 5′GGCTGAAAGTGACTGGGAAACC 3′<br>anti-sense 5′TTGCATTTGGGTCAAACTCC 3′
MMP-3	sense 5′CAGGCTTTCCCAAGCAAATA 3′<br>anti-sense 5′TTGCATTTGGGTCAAACTCC 3′
MMP-9	sense 5′TGTACCGCTATGGTTACACTCG3′<br>anti-sense 5′TGGCTTCCATAGAGTTCCTTCC3′
